# Involvement of A pertussis Toxin Sensitive G-Protein in the Inhibition of Inwardly Rectifying K^+^ Currents by Platelet-Activating Factor in Guinea-Pig Atrial Cardiomyocytes

**DOI:** 10.1155/S0962935194000086

**Published:** 1994

**Authors:** M. Gollasch, T. Kleppisch, D. Krautwurst, D. Lewinsohn, J. Hescheler

**Affiliations:** 1 Physiologisches Institut der Charité Humboldt-Universität zu Berlin Berlin 10115 Germany; 2 Franz-Volhard-Klinik am Max-Delbrück-Centrum für Molekulare Medizin Freie Universität Berlin Berlin 13122 Germany; 3 pharmakologisches Institut Freie Universität Berlin Berlin 14195 Germany; 4 Department of Pharmacology Smooth Muscle Ion Channel Group University of Vermont Medical Research Facility, 55A South Park Drive Colchester VT 14195 Germany

## Abstract

Platelet-activating factor (PAF) inhibits single inwardly rectifying K^+^ channels in guinea-pig ventricular cells. There is currently little information as to the mechanism by which these channels are modulated. The effect of PAF on quasi steady-state inwardly rectifying K^+^ currents (presumably of the I_K1_ type) of auricular, atrial and ventricular cardiomyocytes from guinea-pig were studied. Applying the patch-clamp technique in the whole-cell configuration, PAF (10 nM) reduced the K^+^ currents in all three cell types. The inhibitory effect of PAF occurred within seconds and was reversible upon wash-out. It was almost completely abolished by the PAF receptor antagonist BN 50730. Intracellular infusion of atrial cells with guanine 5′-(*β*-thio)diphosphate (GDPS) or pretreatment of cells with pertussis toxin abolished the PAF dependent reduction of the currents. Neither extracellularly applied isoproterenol nor intracellularly applied adenosine 3′,5′-cyclic monophosphate (cyclic AMP) attenuated the PAF effect. In multicellular preparations of auricles, PAF (10 nM) induced arrhythmias. The arrhythmogenic activity was also reduced by BN 50730. The data indicate that activated PAF receptors inhibit inwardly rectifying K^+^ currents via a pertussis toxin sensitive G-protein without involvement of a cyclic AMP-dependent step. Since I_K1_ is a major component in stabilizing the resting membrane potential, the observed inhibition of this type of channel could play an important role in PAF dependent arrhythmogenesis in guinea-pig heart.


Mediators of Inflammation 3, 45–51 (1994)

### Involvement of a pertussis toxin sensitive G-protein in the inhibition of inwardly rectifying $K^+$ currents by platelet-activating factor in guinea-pig atrial cardiomyocytes

M. Gollasch,[1,2,CA] T. Kleppisch,[1,*] D. Krautwurst,[3] D. Lewinsohn[1] and J. Hescheler[3]

[1]Physiologisches Institut der Charité, Humboldt-Universität zu Berlin, 10115 Berlin; [2]Franz-Volhard-Klinik am Max-Delbrück-Centrum für Molekulare Medizin, Freie Universität Berlin, 13122 Berlin; [3]Pharmakologisches Institut, Freie Universität Berlin, 14195 Berlin, Germany
*Present address: Department of Pharmacology, Smooth Muscle Ion Channel Group, University of Vermont, Medical Research Facility, 55A South Park Drive, Colchester, VT 05446, USA

[CA] Corresponding Author

**PLATELET-activating factor (PAF) inhibits single inwardly rectifying $K^+$ channels in guinea-pig ventricular cells. There is currently little information as to the mechanism by which these channels are modulated. The effect of PAF on quasi steady-state inwardly rectifying $K^+$ currents (presumably of the $I_{K1}$ type) of auricular, atrial and ventricular cardiomyocytes from guinea-pig were studied. Applying the patch-clamp technique in the whole-cell configuration, PAF (10 nM) reduced the $K^+$ currents in all three cell types. The inhibitory effect of PAF occurred within seconds and was reversible upon wash-out. It was almost completely abolished by the PAF receptor antagonist BN 50730. Intracellular infusion of atrial cells with guanine 5′-(β-thio)diphosphate (GDPS) or pretreatment of cells with pertussis toxin abolished the PAF dependent reduction of the currents. Neither extracellularly applied isoproterenol nor intracellularly applied adenosine 3′,5′-cyclic monophosphate (cyclic AMP) attenuated the PAF effect. In multicellular preparations of auricles, PAF (10 nM) induced arrhythmias. The arrhythmogenic activity was also reduced by BN 50730. The data indicate that activated PAF receptors inhibit inwardly rectifying $K^+$ currents via a pertussis toxin sensitive G-protein without involvement of a cyclic AMP-dependent step. Since $I_{K1}$ is a major component in stabilizing the resting membrane potential, the observed inhibition of this type of channel could play an important role in PAF dependent arrhythmogenesis in guinea-pig heart.**

**Key words:** Arrhythmias, G-protein, Inwardly rectifying $K^+$ channels, PAF antagonist, Pertussis toxin, Platelet-activating factor

### Introduction

Platelet-activating factor (PAF, 1-*O*-alkyl-2-(R)acetyl-*sn*-glyceryl-3-phosphocholine) is released from endothelial cells and several types of blood cells in acute allergic and inflammatory reactions (for a review see Snyder[1]). In the whole heart PAF is released under ischaemic conditions.[2] When administered to isolated perfused guinea-pig hearts, PAF reduced the coronary blood flow and exerted negative inotropism.[3–5] The negative inotropic effect was also seen in isolated papillary muscles[6] and auricular preparations of guinea-pig heart.[7]

PAF has also been reported to induce arrhythmias in isolated Langendorff hearts[3,8] and in isolated guinea-pig papillary muscles.[9] In guinea-pig ventricular cardiomyocytes PAF has been shown to inhibit single inwardly rectifying $K^+$ channels.[10] Since the current through this channel type is assumed to stabilize the resting membrane potential and excitability of cardiac cells, the inhibition of inwardly rectifying $K^+$ channels induced by PAF has been suggested to contribute to the genesis of arrhythmias.[10] The recent recognition of the inhibitory effect of PAF on ventricular inwardly rectifying $K^+$ channels prompted us to examine the inhibitory effects of PAF on these currents in cardiomyocytes from various regions of the guinea-pig heart and the possible involvement of G-proteins and cyclic AMP in this process.

### Materials and Methods

*Single cardiomyocytes:* Single cardiomyocytes were prepared by the method previously described by Trube and Hescheler.[11] In brief, adult guinea-pigs of either sex (200–250 g) were anaesthetized with ether. The chest was opened, the heart was rapidly removed and washed twice in Tyrode's solution.[11,12] Retrograde perfusion through the aorta was performed at 37°C using a Langendorff apparatus. The elapsed time from excision of the heart to cannulation and perfusion was less than 1.5 min. The heart was perfused with nominally calcium-free Tyrode's solution ([$Ca^{2+}$], 10–20 μM) at a rate of

© 1994 Rapid Communications of Oxford Ltd

12 ml/min for 5 min. After this period, collagenase (Yakult–Honsha), at a concentration of 0.04%, was added to the calcium-free Tyrode's solution and was recirculated through the heart for 10–20 min. The heart was then removed from the cannula, the atria were separated from the ventricles by cutting, and the auricles were dissected from the atria. Then the auricles, the atria (atria without auricles) and the ventricles were placed into separate flasks and cut into small pieces. Incubation in the collagenase-containing solution proceeded for an additional 5–10 min, during which gentle agitation was provided using a 10 ml pipette. The resulting cell suspensions were filtered through a nylon mesh and centrifuged. The cell pellets were resuspended in 'KB'[12], recentrifuged and washed twice in this solution.

Cells were kept at 6–8°C for 1 to 2 days in 'KB' or modified Tyrode's solution (see below) containing 50% Dulbecco's modified Eagle's medium supplemented with 10% (v/v) horse serum, 4 mM glutamine, 1% (w/v) non-essential amino acids and, if indicated, treated with pertussis toxin (50 ng/ml) for 20–24 h prior to the experiments. For electrophysiological experiments, cells were transferred into a chamber (0.2 ml) and continuously superfused with a modified Tyrode's solution containing (in mM): NaCl 140, $CaCl_2$ 1.8, $MgCl_2$ 1, KCl 5.4, glucose 10, Na–HEPES 10 (pH 7.4, 37°C) at a rate of 5 ml/min (for some details see Gollasch *et al.*[13]). Whole-cell membrane currents were measured according to the method of Hamill *et al.*[14] If not stated otherwise, recording of currents was usually started 5 min after disruption of the membrane patch for intracellular dialysis with the pipette solution. Patch electrodes had an average resistance of 1–2 MΩ when filled with (in mM): K–aspartate 80, KCl 50, $MgCl_2$ 1, Mg–ATP 3, EGTA 10, K–HEPES 10 (pH 7.4). For intracellular infusion of cardiomyocytes with guanine 5'-($\beta$-thio)diphosphate (GDP$\beta$S), the pipette solution contained additionally 100 $\mu$M GDP$\beta$S.[13,15] Quasi steady-state current–voltage relations of inwardly rectifying $K^+$ currents were measured using linear voltage–ramp pulses[16] depolarizing the membrane within 3 s from −100 mV to −40 mV (rate of pulses 0.2 Hz). The method of measurement of steady-state currents through inwardly rectifying $K^+$ channels is subject to small errors which might arise as a result of the existence of overlapping time dependent currents.[17]

For the electrophysiological experiments, only quiescent relaxed auricular, atrial and ventricular cells with clear striations were used. Significant alterations in the slope conductances of the inwardly rectifying $K^+$ currents and in the $\beta$-adrenergic stimulation of $Ca^{2+}$ currents during storage of the cells, were not observed.

*Isolated auricular muscle:* Auricular muscles dissected from left atria were prepared from guinea-pigs anaesthetized with ether as described previously.[7] The muscles were superfused with gassed (95% $O_2$ and 5% $CO_2$) Tyrode's solution composed of (in mM): NaCl 136.9, KCl 2.7, $NaHCO_3$ 22.4, $CaCl_2$ 1.8, $MgCl_2$ 1, $NaH_2PO_4$ 0.5, glucose 5.6 (pH 7.3–7.4 at 37°C). The flow rate was 7 ml/min. The muscles were paced at 0.5 Hz by square wave pulses (duration 1 ms; voltage was adjusted to 1.5-fold about threshold) and isometric contractions were measured using a force transducer type 316 with RCA-5734 (Hugo Sachs Elektronik, Freiburg, Germany), as described previously.[7] Data were analysed off-line using a program written by D. Lewinsohn.

*Materials:* PAF ($C_{16}$) was obtained from Serva (Heidelberg, Germany). Collagenase and guanine 5'-($\beta$-thio)diphosphate (GDP$\beta$S) were purchased from Yakult–Honsha (Tokyo, Japan) and Calbiochem (Bad Soden, Germany), respectively. BN 50730 and pertussis toxin were kindly provided by Dr P. Braquet (IHB Research Labs, Le Plessis, France) and Dr M. Yajima (Kyoto, Japan), respectively. Glutamine and non-essential amino acids were purchased from Biochrom (Berlin, Germany); horse serum was from GIBCO (Paisley, UK).

PAF was dissolved in saline containing 0.25% bovine serum albumin. BN 50730 (P. Braquet and J. Escanu) was dissolved in a stock solution of 1:2 dimethylsulfoxide (DMSO):100% ethanol at 4 mg/ml. Immediately before application, an equal volume of 0.2 N HCl was added and this was diluted in 0.9% NaCl. The final concentrations of DMSO and ethanol had no effect on inwardly rectifying $K^+$ currents and on contractions.

*Statistics:* All values are mean ± S.E.M.; *n* represents the number of cells examined. Differences between means were tested for statistical significance by the Wilcoxon rank sum test or Mann–Whitney–Wilcoxon on test. $p < 0.05$ was considered significant.

### Results

*Effect of PAF on inwardly rectifying $K^+$ currents:* Inwardly rectifying $K^+$ currents were studied in auricular, atrial and ventricular cardiomyocytes from guinea-pigs (Fig. 1). In 5.4 mM extracellular $K^+$, currents reversed at $-71 \pm 3$ mV ($n = 5$), $-76 \pm 3$ mV ($n = 8$) and $-78 \pm 3$ mV ($n = 5$), respectively. As required for a current mainly carried by $K^+$, the reversal potential ($E_{rev}$) shifted when cells were exposed to different levels of external $K^+$. $E_{rev}$ was found to be $-63 \pm 4$ mV ($n = 4$) and $-51 \pm 4$ mV in atrial cells and

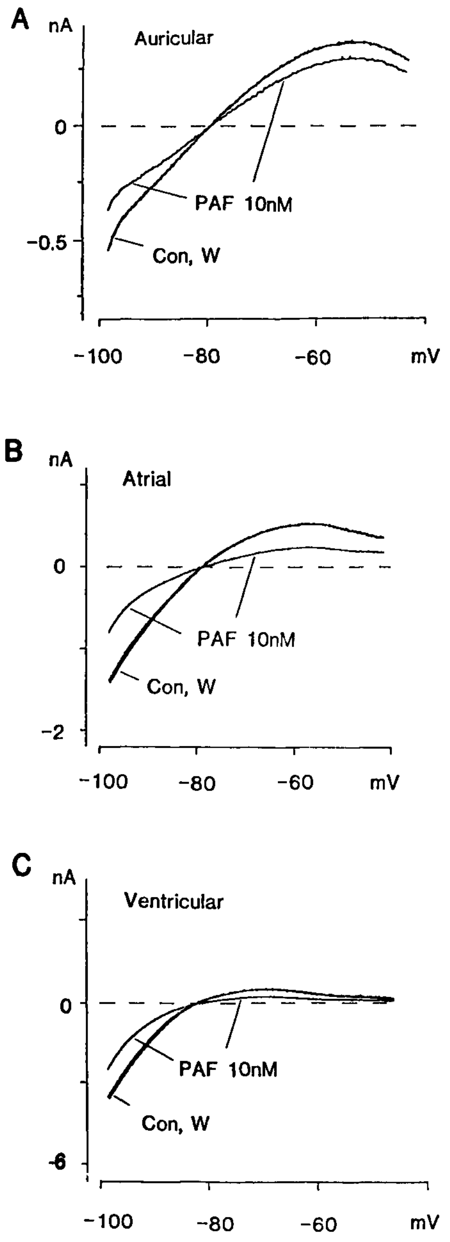

FIG. 1. Effect of PAF on inwardly rectifying $K^+$ conductance in auricular (Panel A), atrial (Panel B) and ventricular (Panel C) cells. Cells were held at −80 mV, and linear voltage–ramp pulses of 20 mV/s were applied. Con, control current–voltage relationship; PAF, current–voltage relationship in the presence of PAF; W, current–voltage relationship after washout of PAF. Dashed lines represent zero currents.

−66 ± 5 mV ($n = 4$) and −53 ± 6 mV ($n = 3$) in ventricular cells using 11 mM and 22 mM external $K^+$, respectively. At 2 mM $K^+$ outside, the current reversed at a potential below −100 mV. The current was further characterized by its inward rectification; the slope conductance was large below the reversal and declined at more positive potentials. Currents could be blocked by $Ba^{2+}$ (10 mM) or $Cs^+$ (5 mM) (see Fig. 2 for auricular cells; not shown for atrial and ventricular cells but see Bechem *et al.*[18]). Extracellular application of $Ca^{2+}$ channel blockers $Cd^{2+}$ (1 mM) or (±)-isradipine (1 μM) did not modify the currents. The inwardly rectifying $K^+$ currents represent the most likely currents through $I_{K1}$ channels, since the electrophysiological and pharmacological properties correspond to the $I_{K1}$ channel type extensively studied in atrium and ventricle.[11,19]

PAF (10 nM) reduced inwardly rectifying currents (Figs 1 and 3); the slope conductance measured at the zero current potential declined from −23 ± 2 nS to −18 ± 2 nS ($n = 5$) in auricular and from −64 ± 4 nS to −35 ± 3 nS ($n = 6$) in atrial cells. Between −95 and −90 mV the slope conductance was reduced from −24 ± 6 nS to −19 ± 3 nS in auricular cells and from −77 ± 8 nS to −48 ± 9 nS in atrial cells. In ventricular cells the slope conductance measured at the zero current potential was decreased by PAF from −90 ± 6 nS to −58 ± 9 nS; between −95 and 90 mV it was reduced from −272 ± 19 nS to −237 ± 13 nS ($n = 5$). $E_{rev}$ was not significantly reduced in atrial, auricular and ventricular cells (5 ± 7 mV ($n = 6$), 3 ± 6 mV ($n = 5$) and 3 ± 4 mV ($n = 5$) depolarization, respectively). The inhibitory effect of PAF on $I_{K1}$ occurred within seconds and was fully reversible after removal from the bath (Fig. 4). On further applications (up to four times during the superfusion period of approximately 15 min), the inhibition was of reproducible amplitude (not shown).

In order to demonstrate the involvement of PAF receptors in the inhibition of the currents, the specific PAF receptor antagonist BN 50730 was used.[20–22] Whereas external application of BN 50730 (1 μM) did not affect control currents of atrial cells, the inhibitory effect of PAF (10 nM) on the current was almost completely abolished in the presence of BN 50730 (1 μM) (Fig. 4). In five cells the mean attenuation of inwardly rectifying currents by PAF (10 nM) plus BN 50730 (1 μM) was −3 ± 3%. As demonstrated in Fig. 4 (points d–f in panel B), the blockage of the PAF (10 nM) effect by BN 50730 (1 μM) occurred quickly. To block the effect of 1 μM PAF, BN 50730 had to be administered at a concentration of 10 μM.

*Involvement of GTP-binding proteins:* The PAF receptor interacts with its effectors through GTP-binding (G) proteins.[1] Therefore, we tested whether the PAF effect on the $K^+$ current is sensitive to GDPβS, (100 μM), a GDP analogue which stabilizes all G-proteins in their inactive form. Intracellular infusion of GDPβS for 2–3 min via the patch-pipette only marginally affected the PAF dependent inhibition of the current (Fig. 5). A significant reduction of the PAF effect was seen after longer periods of cell dialysis with GDPβS. After 5–7 min cell dialysis PAF induced a current inhibition of 11 ± 9% ($n = 5$); after 10–15 min the current was inhibited by 10 ± 8% ($n = 3$, Fig. 5).

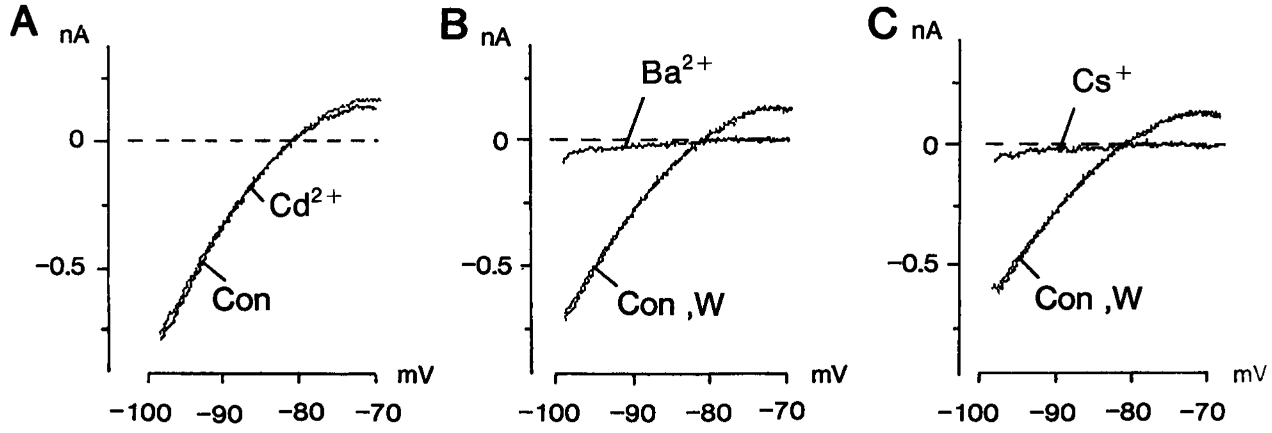

FIG. 2. Effect of $Cd^{2+}$, $Ba^{2+}$ and $Cs^{+}$ on inwardly rectifying $K^{+}$ currents in cells isolated from guinea-pig auricular muscle. Cells were held at −80 mV, and linear voltage–ramp pulses of 20 mV/s were applied. Con, control current–voltage relationships; $Cd^{2+}$ (Panel A), $Ba^{2+}$ (Panel B) and $Cs^{+}$ (Panel C), current–voltage relationships in the presence of 1 mM $Cd^{2+}$, 10 mM $Ba^{2+}$ and 5 mM $Cs^{+}$, respectively; W, current–voltage relationships after removal of PAF from the bath. Dashed lines represent zero currents.

This is in line with the calculated diffusion velocity of GDP$\beta$S (mol. wt 477) through the pipette opening.[15]

The involvement of a G-protein was further

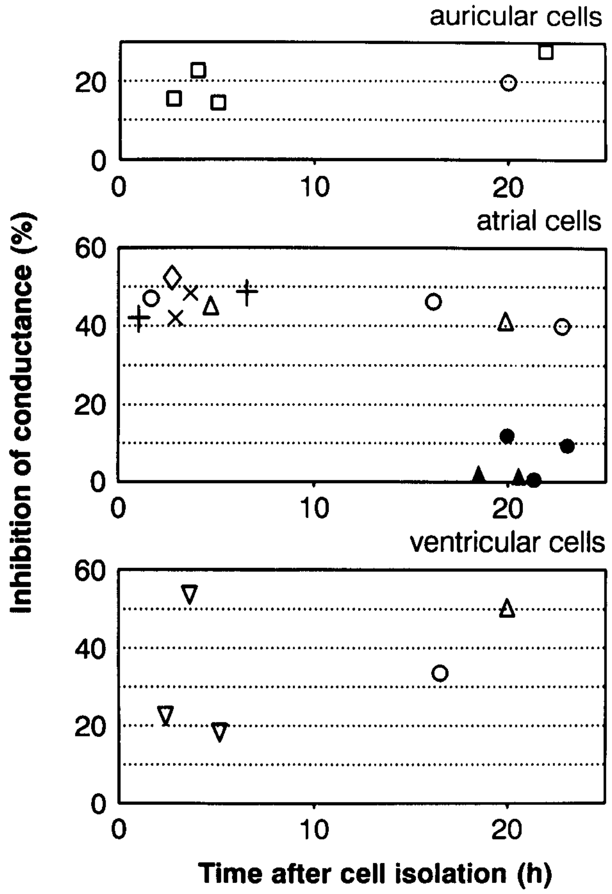

FIG. 3. Inhibition of inwardly rectifying $K^{+}$ currents by PAF in auricular, atrial and ventricular guinea-pig cardiomyocytes. The figure shows the percentage of inhibition of the slope conductance of inwardly rectifying $K^{+}$ currents by PAF (10 nM) in untreated cells (open symbols, × and +) and in cells treated with pertussis toxin (filled symbols, 50 ng/ml) at different times after enzymatic isolation of cardiomyocytes. The slope conductance was determined at zero current potential. Identical forms of symbols indicate that the cells were obtained from the same hearts. Cardiomyocytes obtained from hearts × and + were intracellularly infused with 100 $\mu$M cAMP (intracellular dialysis, 5–10 min).

confirmed by pretreatment of cells with pertussis toxin (PT). This exotoxin from *Bordetella pertussis* is known to add ADP–ribosyl to the $\alpha$-subunits of the $G_i$- as well as $G_o$- like G-proteins, thus preventing the coupling of hormone activated receptors to G-proteins.[23] In atrial cells pretreated with PT, PAF (10 nM) failed to inhibit the $K^{+}$ current (Figs 3 and 5); the mean modulation of the current was $-4 \pm 4\%$ ($n = 5$).

PT sensitive G-proteins are known to couple receptors with the effector enzyme adenylyl

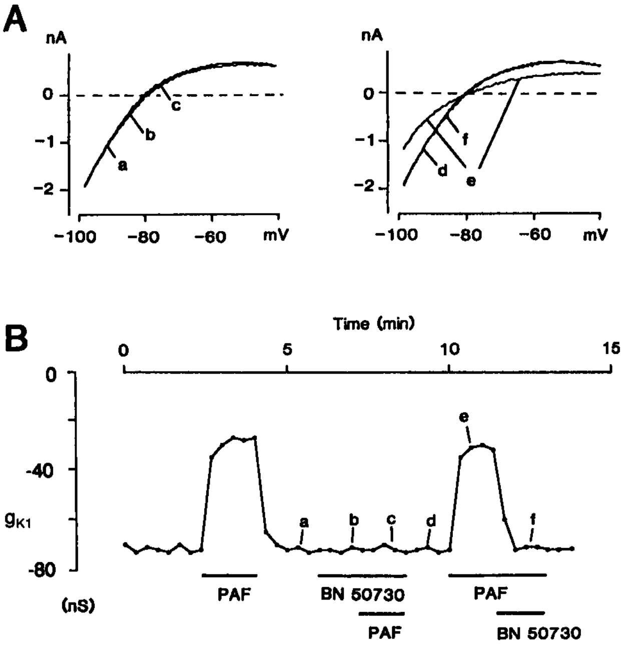

FIG. 4: Effect of BN 50370 on the PAF dependent inhibition of inwardly rectifying $K^{+}$ current. The atrial cell was held at −80 mV, and linear voltage–ramp pulses of 20 mV/s were applied. Panel A: superimposed current–voltage relationships (a–f) defined in the time course of the experiment shown in Panel B. The presence of PAF (10 nM) and BN 50730 (1 $\mu$M) in the bath is indicated by horizontal bars. $g_{K1}$, slope conductance of the inwardly rectifying $K^{+}$ current determined at −80 mV. Dashed lines represent zero currents. After wash-out of BN 50370 and PAF, the subsequent addition of PAF (10 nM) to the bath caused an inhibition of $g_{K1}$ comparable to those of the previous PAF applications (not shown).

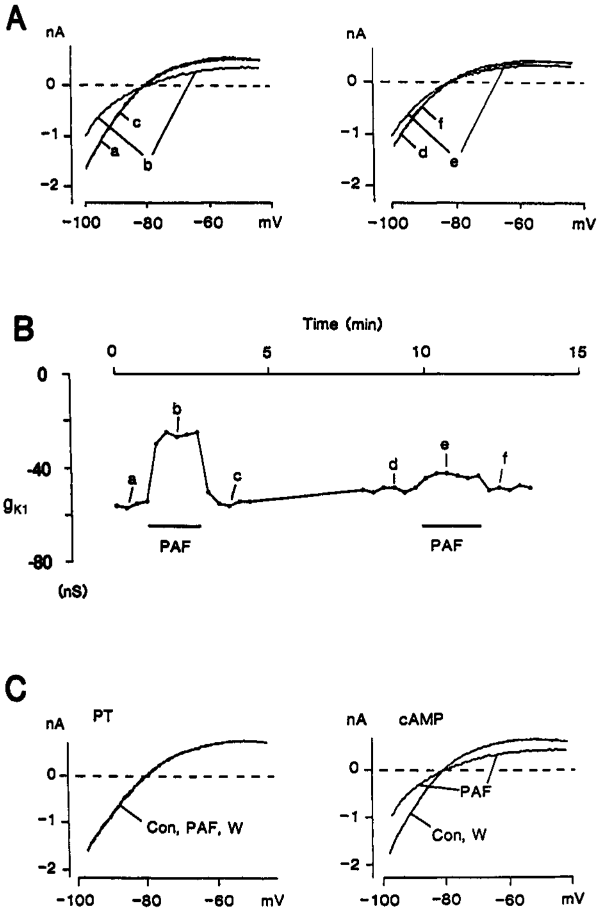

FIG. 5. Effects of GDP$\beta$S, pertussis toxin and cAMP on the PAF dependent inhibition of inwardly rectifying $K^+$ current. Atrial cells were held at −80 mV, and linear voltage–ramp pulses of 20 mV/s were applied. Panel A: superimposed current–voltage relationships (a–f) defined in the time course of the experiment shown in Panel B. Recording of currents was started 30 s after disruption of the membrane for intracellular dialysis with pipette solution containing 100 $\mu$M GDP$\beta$S. The presence of PAF (10 nM) in the bath is indicated by horizontal bars. $g_{K1}$, slope conductance of the inwardly rectifying $K^+$ current determined at −80 mV. Panel C: shows the current–voltage relationships recorded from cells pretreated with pertussis toxin (PT, 50 ng/ml, left part of the panel) and intracellularly dialysed with pipette solution containing 100 $\mu$M cAMP for 8 min prior to the experiment (right part of the panel). Con, control current–voltage relationships before application of PAF; PAF, current–voltage relationships in the presence of PAF (10 nM); W, current–voltage relationships after removal of PAF from the bath. Dashed lines represent zero currents.

cyclase.[24] In endothelial cells and platelets it has been demonstrated that PAF receptors inhibit the adenylyl cyclase.[25,26] To test whether such a mechanism is involved in the inhibition of inwardly rectifying $K^+$ currents, a possible involvement of adenosine 3′,5′-cyclic monophosphate (cyclic AMP) in the regulation of the current was examined. It was found that direct intracellular application of cyclic AMP (100 $\mu$M in the pipette solution) had no effect on the $K^+$ current during 5–10 min infusion and did not prevent the PAF dependent reduction of the current in atrial cells ($n = 4$, see Figs 3 and 5).

*Arrhythmogenic activity of PAF:* In isolated left guinea-pig auricle muscles, contractile responses were observed for about 10 min prior to each experiment. During this period, no extrasystoles were seen; the interval between electrically stimulated beats remained stable at 2 s; no extra beats were observed.

PAF (10 nM, 10–15 min superfusion time) induced rhythm disturbances in nine out of 15 muscles studied. The rhythm disturbances induced by PAF occurred without detectable delay after PAF application and were equally distributed during the whole superfusion period of PAF. The interval from beat-to-beat in the muscles with PAF induced arrhythmic activity was reduced to 0.6 ± 0.4 s; 86.4 ± 37.6 extra beats per min were detected during the PAF application ($n = 9$, 10 min of PAF superfusion). The amplitude of contraction was on average reduced by PAF to 61 ± 7% of control values (see also Gollasch *et al.*[7]).

BN 50730 (1 $\mu$M) inhibited the PAF induced rhythm disturbances. In the nine muscles with PAF induced arrhythmic activity the PAF receptor antagonist increased the interval from beat-to-beat to 1.9 ± 0.2 s; 5.1 ± 1.8 extra beats per min were observed during superfusion of the drugs. When BN 50730 (1–10 $\mu$M) was applied 10 min prior to application of PAF (10 nM), no arrhythmias were detected within 20 min superfusion of the drugs ($n = 4$, see also Koltai *et al.*[22]). The PAF-reduced amplitude of contraction was restored by BN 50730 to 90 ± 12% of control values.

### Discussion

Evidence is presented that PAF inhibits inwardly rectifying $K^+$ currents (presumably of the $I_{K1}$-type) in cardiomyocytes from various regions of guinea-pig heart. Since the effect of PAF occurred at low concentrations of PAF (nanomolar range) and was fully antagonized by BN 50730, presumably it is mediated by specific cardiac receptors for PAF.[10]

As shown in other cellular systems the PAF receptor(s) couples to its effector via G-proteins.[1] It was found that the inhibitory effect of PAF on the inwardly rectifying $K^+$ current was suppressed by GDP$\beta$S as well as pertussis toxin (PT). These results suggest the involvement of a PT sensitive G-protein in the receptor-induced reduction of the currents. In heart, G-protein dependent regulation of various types of $K^+$ channels has been described. PT sensitive $G_K$-proteins ($G_{i1}$, $G_{i2}$ and $G_{i3}$) are involved in the muscarinic acetylcholine receptor dependent activation of the $K_{ACh}$ current in atrial cells. The ATP sensitive $K^+$ channel ($K_{ATP}$) is directly activated by PT-sensitive $G_{i3}$ proteins; pacemaker $K^+$ channels ($I_f$) are activated by $\beta$-adrenoceptors via $G_s$-proteins and inhibited by muscarinic agonists via PT sensitive $G_{o(i)}$ proteins (for review see Brown and Birnbaumer[23]).

The data from Wahler *et al.*[10] support the view that the PAF-induced inhibition of inwardly rectifying $K^+$ currents is mediated by intracellular messengers. These authors observed an inhibition of single inwardly rectifying $K^+$ channels ($i_{K1}$) in cell-attached patches following application of 1 nM PAF to the bath. Under these conditions the decrease of channel activity occurred with a delay of 7–10 min. In contrast, whole-cell inwardly rectifying $K^+$ currents were reduced within 10–20 s by PAF at 10 nM (present data); 1 nM PAF decreased the current after a delay of 0.5–1.5 min (M.G., unpublished). The faster effect of PAF on whole-cell currents may be explained by usage of the whole-cell clamp configuration which favours the ready diffusion of intracellular messengers from the PAF receptor to the channel protein.

Possible candidate messengers are products generated by phospholipase $A_2$ ($PLA_2$) which is activated via PAF receptors in various cell types.[1] Whereas the hormonal activation of phospholipase C in most cell types, including cardiomyocytes, is insensitive to PT, activation of $PLA_2$, e.g. by $\alpha_1$-adrenoceptor stimulation, may be sensitive to the toxin.[27] The stimulation of $\alpha_1$-adrenoceptors causes a PT sensitive decrease of inwardly rectifying $K^+$ current ($I_{K1}$) in canine Purkinje fibres.[28]

Nakajima *et al.*[29] reported a PT sensitive mechanism for activation of guinea-pig atrial $K_{ACh}$ channels by PAF at micromolar concentrations probably being mediated by arachidonic acid metabolites (under GTP conditions). An activation of muscarinic $K^+$ current by PAF ($>0.2\ \mu M$) was also observed in bullfrog atrial myocytes.[30] The authors, however, found that inhibition of 5- and 12-lipoxygenases by eicosatetranoic acid did not prevent the PAF-mediated increase in muscarinic $K^+$ current. Therefore, Ramos-Franco *et al.*[30] suggested that the mechanism of PAF action on bullfrog muscarinic $K^+$ current is analogous to acetylcholine in that PAF binds to its (low-affinity) receptor, which is coupled to a G-protein (possibly $G_{i1-3}$), which in turn activates $K_{ACh}$ channels.

It has been suggested that subtypes of PAF receptors exist.[1] Both high-affinity ($K_d$ in the lower nM range) and low-affinity ($K_d$, 10–500 nM or more) receptors for PAF have been described. The involvement of low-affinity binding sites in PAF dependent activation of muscarinic $K^+$ current was suggested by the high PAF concentrations (micromolar range) used in the studies of Nakajima *et al.*[29] and Ramos-Franco *et al.*[30] PAF at concentrations below 0.2 $\mu$M did not increase $K_{ACh}$.[29,30]

The presence of PAF receptors with high-affinity binding sites in cardiomyocytes mediating the inhibition of inwardly rectifying $K^+$ currents can be suggested by the PAF concentrations (nanomolar range) used in this and in the study of Wahler *et al.*[10] High concentrations of PAF (micromolar range) also induced an inhibition of the current. Therefore, one might suggest that the inhibition of cardiac inwardly rectifying $K^+$ ($i_{K1}$) currents is mediated by a PAF receptor subtype different from that stimulating atrial $K_{ACh}$ channels.

The initiation of cardiac excitation in working myocardium depends on the threshold to generate an action potential, which by itself is determined by $I_{K1}$.[31] A large $I_{K1}$ implies that the resting potential is 'stabilized', i.e. stronger depolarizing currents are necessary to trigger an action potential.[31] Data obtained with low concentrations of $Ba^{2+}$ suggest that the inhibition of inwardly rectifying $K^+$ channels is causally related to arrhythmogenesis in ventricular fibres.[32] By analogy, the PAF dependent inhibition of inwardly rectifying $K^+$ currents may contribute to the genesis of observed arrhythmias and automaticity in multicellular cardiac preparations. Indeed, PAF induces rhythm disturbances in auricular multicellular preparations and also inhibits inwardly rectifying $K^+$ currents in cardiomyocytes isolated from supraventricular regions of the heart at the same range of concentration.

With regard to the previous literature, the observed inhibition of inwardly rectifying $K^+$ currents is certainly not the only effect of PAF on cardiomyocytes. At low concentrations (nanomolar range) PAF augments $Ca^{2+}$ currents in multicellular guinea-pig preparations.[6,7,33] At high concentrations (micromolar range) PAF has been demonstrated to decrease intracellular $Na^+$ activity,[34] to reduce $Ca^{2+}$ currents and to stimulate an outward current presumably through delayed outward $K^+$ channels in frog atrial trabeculae.[7] Although these mechanisms might also play a role in PAF induced arrhythmogenesis, they would act synergistically to cause arrhythmias in multicellular preparations.

Numerous questions about the processes involved in PAF induced arrhythmogenesis in heart muscle still remain (modulation of other ion transport systems, influence of endothelium etc.). Nevertheless, it is felt confidently that the hypotheses on the involvement of PAF inhibited $I_{K1}$ in arrhythmogenesis[10] will be useful to guide this work.

### References

1. Snyder F. Platelet-activating factor and related acetylated lipids as potent biologically active cellular mediators. *Am J Physiol* 1990; **259**: C697–C708.
2. Montrucchio G, Alloatti G, Tetta C, *et al.* Release of platelet-activating factor from ischemic-reperfused rabbit heart. *Am J Physiol* 1989; **256**: H1236–1246.
3. Levi R, Burke JA, Guo ZG, *et al.* Acetyl glyceryl ether phosphorylcholine (AGEPC). A putative mediator of cardiac anaphylaxis in the guinea pig. *Circ Res* 1984; **54**: 117–124.
4. Viossat I, Chapelat M, Chabrier PE, Braquet P. Effects of platelet activating factor (PAF) and its receptor antagonist BN 52021 on isolated perfused guinea-pig heart. *Prostaglandins Leukotrienes and Essential Fatty Acids* 1989; **38**: 189–194.
5. Felix SB, Steger A, Baumann G, Busch R, Ochsenfeld G, Berdel WE. Platelet-activating factor-induced coronary constriction in the isolated perfused guinea pig heart and antagonistic effects of the PAF antagonist WEB 2086. *J Lipid Med* 1990; **2**: 9–20.

6. Camussi G, Alloatti G, Montrucchio G, Meda M, Emanuelli G. Effect of platelet activating factor on guinea-pig papillary muscle. *Experientia* 1984; **40**: 697–699.
7. Gollasch M, Ignatieva V, Kobrinsky E, Vornovitsky E, Zaborovskaya L. Electrophysiological mechanisms responsible for the action of PAF in guinea-pig myocardium. *J Lipid Mediat* 1991; **3**: 139–159.
8. Koltai M, Tosaki A, Hosford D, Braquet P. Ginkgolide B protects isolated hearts against arrhythmias induced by ischemia but not reperfusion. *Eur J Pharmacol* 1989; **164**: 293–302.
9. Shigenobu K, Mori T, Kamata K, Kasuya Y. Platelet-activating factor: lack of direct action on guinea pig myocardium and possible transmitter release from cardiac sympathetic nerve endings at high concentrations. *Can J Physiol Pharmacol* 1989; **67**: 669–674.
10. Wahler GM, Coyle DE, Sperelakis N. Effects of platelet-activating factor on single potassium channel currents in guinea pig ventricular myocytes. *Molec Cell Biochem* 1990; **93**: 69–76.
11. Trube G, Hescheler J. Inward-rectifying channels in isolated patches of the heart cell membrane: ATP-dependence and comparison with cell-attached patches. *Pflügers Arch* 1984; **401**: 178–184.
12. Isenberg G, Klöckner U. Calcium tolerant ventricular myocytes prepared by pre-incubation in a "KB" medium. *Pflügers Arch* 1982; **395**: 6–18.
13. Gollasch M, Hescheler J, Spicher K, Klinz F-J, Schultz G, Rosenthal W. Inhibition of $Ca^{2+}$ channels via $\alpha_2$-adrenergic and muscarinic receptors in pheochromocytoma (PC-12) cells. *Am J Physiol* 1991; **260**: C1282–C1289.
14. Hamill OP, Marty A, Neher E, Sakmann B, Sigworth FJ. Improved patch-clamp techniques for high-resolution current recording from cells and cell-free membrane patches. *Pflügers Arch* 1981; **391**: 85–100.
15. Hescheler J, Kameyama M, Speicher R. Internal patch-pipette perfusion. In: Kettenmann H, Grantyn R, eds. *Practical Electrophysiological Methods: A guide for* in vitro *studies in vertebrate neurobiology*. Wiley-Liss, Inc: New York, 241–248.
16. Kiyosue T, Arita M. Effects of lysophosphatidylcholine on resting potassium conductance of isolated guinea pig ventricular cells. *Pflügers Arch* 1986; **406**: 296–302.
17. Hume JR, Uehara A. Ionic basis of the different action potential configurations of single guinea-pig atrial and ventricular myocytes. *J Physiol* 1984; **368**: 525–544.
18. Bechem M, Glitsch HG, Pott L. Properties of an inward rectifying K channel in the membrane of guinea-pig atrial cardioballs. *Pflügers Arch* 1983; **399**: 186–193.
19. Harvey RD, Ten Eick. On the role of sodium ions in the regulation of the inward-rectifying potassium conductance in cat ventricular myocytes. *J Gen Physiol* 1989; **94**: 329–348.
20. Braquet P, Laurent JP, Rolland A, Martin C, Pommier J, Hosford D, Escanu A. From ginkgolides to N-substituted piperidinothieno diazepines, a new series of highly potent dual antagonists. *Advances in Prostaglandin, Thromboxane, and Leukotriene Research* 1990; **21**: 929–937.
21. Dyson MC, Bellan JA, Minkes RK, *et al.* Influence of SKF 95587 and BN 50730 on bronchoconstrictor responses in the cat. *J Pharmacol Exp Ther* 1990; **255**: 1320–1327.
22. Koltai M, Tosaki A, Hosford D, Esanu A, Braquet P. Effect of BN 50739, a new platelet activating factor antagonist, on ischemia induced ventricular arrhythmias in isolated working rat hearts. *Cardiovascular Res* 1991; **25**: 391–397.
23. Brown AM, Birnbaumer L. Ionic channels and their regulation by G protein subunits. *Ann Rev Physiol* 1990; **52**: 197–213.
24. Drummond GE, Severson DL. Cyclic nucleotides and cardiac function. *Circ Res* 1979; **44**: 145–152.
25. Haslam RJ, Vanderwel MJ. Inhibition of platelet adenylate cyclase by 1-O-alkyl-2-O-acetyl-*sn*-glyceryl-3-phosphorylcholine (platelet-activating factor). *J Biol Chem* 1982; **257**: 6879–6885.
26. Grigorian GY, Ryan US. Platelet-activating factor effects on bovine pulmonary artery endothelial cells. *Circ Res* 1987; **61**: 389–395.
27. Axelrod J, Burch RM, Jelsema CL. Receptor-mediated activation of phospholipase $A_2$ via GTP-binding proteins: arachidonic acid and its metabolites as second messengers. *Trends Neuro Sci* 1988; **11**: 117–123.
28. Shah A, Cohen IS, Rosen MR. Stimulation of cardiac $\alpha$ receptors increases Na/K pump and decreases $g_K$ via a pertussis toxin-sensitive pathway. *Biophys J* 1988; **54**: 219–225.
29. Nakajima T, Sugimoto T, Kurachi Y. Platelet-activating factor activates $G_K$ via arachidonic acid metabolites. *FEBS Lett* 1991; **289**: 239–243.
30. Ramos-Franco J, Lo F, Breitweiser G. Platelet-activating factor receptor-dependent activation of the muscarinic $K^+$ current in bullfrog atrial myocytes. *Circ Res* 1993; **72**: 786–794.
31. Trautwein W. Membrane currents in cardiac muscle fibres. *Physiol Rev* 1973; **53**: 793–835.
32. Malecot C, Coraboeuf E, Coulombe A. Automaticity of ventricular fibres induced by low concentrations of barium. *Am J Physiol* 1984; **247**: H429–H439.
33. Tamargo J, Tejerina T, Delgado C, Barrigon S. Electrophysiological effects of platelet-activating factor (PAF-acether) in guinea-pig papillary muscles. *Eur J Pharmacol* 1985; **109**: 219–227.
34. Robertson DA, Wang DY, Lee CO, Levi R. Negative inotropic effect of platelet-activating factor: association with a decrease of intracellular sodium activity. *J Pharmacol Exp Ther* 1988; **245**: 124–128.

ACKNOWLEDGEMENTS. The authors thank Drs E. Schubert, J. Patlak and L. Linke for helpful dicussions and Drs G. Schultz, M. Nelson, J. Quayle, G. Trube, and M. Kameyama for their comments on a first version of the manuscript. Drs P. Braquet and A. Esanu kindly supplied the BN 50730. The expert technical assistance of I. Dichtl, M. Bigalke and W. Stamm is also acknowledged. This work was supported by a grant of the Deutsche Forschungsgemeinschaft to G. Schultz and of the Bundesministerium für Forschung und Technik to M. Gollasch.

**Received 6 August 1993; accepted 19 November 1993**
